# Bacteria Stimulate Hatching of Yellow Fever Mosquito Eggs

**DOI:** 10.1371/journal.pone.0024409

**Published:** 2011-09-06

**Authors:** Loganathan Ponnusamy, Katalin Böröczky, Dawn M. Wesson, Coby Schal, Charles S. Apperson

**Affiliations:** 1 Department of Entomology, North Carolina State University, Raleigh, North Carolina, United States of America; 2 Department of Tropical Medicine, Tulane University, New Orleans, Louisiana, United States of America; French National Centre for Scientific Research - Université Aix-Marseille, France

## Abstract

**Background:**

*Aedes aegypti* Linnaeus is a peridomestic mosquito that lays desiccation-resistant eggs in water-filled human-made containers. Previous investigations connected egg hatching with declining dissolved oxygen (DO) that is associated with bacterial growth. However, past studies failed to uncouple DO from other potential stimulatory factors and they contained little quantitative information about the microbial community; consequently, a direct role for bacteria or compounds associated with bacteria in stimulating egg hatching cannot be dismissed.

**Methodology/Principal Findings:**

Environmental factors stimulating hatch of *Ae. aegypti* eggs were investigated using non-sterile and sterile white oak leaf (WOL) infusions and a bacterial culture composed of a mix of 14 species originally isolated from bamboo leaf infusion. In WOL infusion with active microbes, 92.4% of eggs hatched in 2-h at an average DO concentration of 2.4 ppm. A 24-h old bacterial culture with a DO concentration of 0.73 ppm also stimulated 95.2% of eggs hatch within 1-h. In contrast, only 4.0% of eggs hatched in sterile infusion, whose DO averaged 7.4 ppm. Effects of bacteria were uncoupled from DO by exposing eggs to bacterial cells suspended in NaCl solution. Over a 4-h exposure period, 93.8% of eggs hatched while DO concentration changed minimally from 7.62 to 7.50 ppm. Removal of bacteria by ultra-filtration and cell-free filtrate resulted in only 52.0% of eggs hatching after 4-h at an average DO concentration of 5.5 ppm.

**Conclusions/Significance:**

Collectively, the results provide compelling evidence that bacteria or water-soluble compounds secreted by bacteria, not just low DO concentration, stimulate hatching of *Ae. aegypti* eggs. However, the specific cues involved remain to be identified. These research findings contribute new insight into an important aspect of the oviposition biology of *Ae. aegypti*, a virus vector of global importance, providing the basis for a new paradigm of environmental factors involved in egg hatching.

## Introduction

*Aedes aegypti* Linnaeus is the primary vector of dengue and dengue hemorrhagic fever throughout the world [1]. These illnesses are the most prevalent vector-borne viral diseases of humans worldwide with an estimated 50 million infections per year [2]. *Aedes aegypti*, a peridomestic mosquito, deposits eggs on the sides of water-filled human-made containers just above the water line and directly on the surface of the water [3]. Embryogenesis is completed within several days after oviposition, but embryos can withstand desiccation, and do not hatch for up to one year without proper hatching stimuli [4].

Several biological and environmental cues that stimulate egg hatch in mosquitoes have been studied. In early experiments, live microbial cultures were shown to stimulate eggs of *Ae. aegypti* to hatch. However, no hatching was observed with heat-killed microbial cultures or filter-sterilized culture media, suggesting that sterilization eliminated the hatching cues [5], [6]. When floodwater mosquito eggs (*Ae. vexans* (Meigen) and *Ae. aldrichi* Dyar and Knab [ =  *Ae*. *sticticus* (Meigen)]) were flooded with an actively fermenting plant infusion, significant egg hatch was observed, which was thought to be due to the presence of amino acids, proteins, and phosphate salts in the vegetation [7]. Subsequently, it was reported that bacterial species cultured from the plant infusion were differentially active in achieving egg hatch [8].

Gillett et al. [9] re-examined the asynchronous hatching of cohorts of *Ae. aegypti* eggs with the intent of defining causal factors. Their study suggested that eggs bearing greater numbers of surface bacteria were the first to hatch. Furthermore, they found that mosquito larvae removed bacteria from the egg surface by grazing, and thus delayed egg hatch. Research completed with the eastern treehole mosquito *Ae*. *triseriatus* (Say) [10] corroborated the findings of Gillett et al. Hatch of *Ae*. *triseriatus* eggs was suppressed as larval densities in experimental microcosms increased. Moreover, nutrient broth has been used to stimulate egg hatch of *Aedes* spp. by promoting the rapid growth of microbial populations [11], [12], [13].

Reduction of dissolved oxygen (DO) by chemical or biological factors has been associated with egg hatching [8], [12], [14] and this effect has led to the currently-accepted hypothesis that DO concentration is the principal factor regulating egg hatch. However, there is no direct experimental evidence that declining DO is the sole factor involved in stimulating egg hatch under natural or laboratory conditions. Particularly, past investigations involving microbes have failed to uncouple changes in DO concentration from other putative stimulatory factors, such as bacteria or bacterial metabolites. These studies have provided little quantitative information about the microbial populations, such as the cell densities used in experiments, and DO concentration has often not been measured.

The objective of the present study was to investigate the role of bacteria and bacteria-associated compounds in stimulating eggs of *Ae*. *aegypti* to hatch. More specifically, we addressed the following questions. Does a microbe-free plant infusion stimulate egg hatching in *Ae. aegypti*? Can microbial cells transferred to a microbe-free plant infusion stimulate egg hatching? Does the bacteria-free filtrate of a bioactive bacterial culture contain egg-hatching stimulants? Can bacterial cells that were removed from a 24 h old bacterial culture by centrifugation and transferred to physiological saline solution stimulate egg hatch under elevated DO conditions?

## Materials and Methods

### Mosquito colony and collection of eggs for hatching experiments

A laboratory colony of *Aedes aegypti* was established from field-collected eggs from New Orleans, LA, USA, in 2003. Mosquitoes were reared as previously described [15], [16]. Females were blood-fed on a forearm of a co-author of the manuscript (CSA) with written informed consent. This activity has been reviewed and approved by the NCSU Biosafety Committee (Registration #2010-040421). Blood-fed females of the F_2-3_ generation were placed in cages and provided with a 10% sucrose solution *ad libitum*. On the 4^th^ or 5^th^ day after blood feeding, black plastic cups containing sterilized strips of seed germination paper (Anchor Paper Co., St. Paul, MN, USA) and sterilized water were placed in the cages to collect eggs. After an exposure period of 2-days, the seed germination papers were removed, air-dried for several min and covered with Press'N Seal® plastic wrap (The Glad Products Co., Oakland, CA). Eggs were stored on seed germination paper in a desiccation cabinet at room temperature and 95% RH. Eggs were used in experiments 10–20 days after they were collected.

### Egg hatching bioassays

With a fine camel hair brush, eggs were carefully transferred from seed germination paper into a dry watch glass (3 cm dia.×2 cm high) placed on a mini light box fitted with a mini magnifier (Scienceware, Pequannock, NJ, USA). Each watch glass contained 50 eggs and was regarded an experimental replicate. Eggs were distributed evenly over the bottom of each watch glass. Seven mL of an experimental solution (infusion, bacterial culture, or cell suspension) or a control solution (sterile R2A medium, saline, or water) were carefully added to the watch glass, submerging the eggs. Hatched eggs were counted with the aid of a light box and magnifier immediately before eggs were submerged (0-h) and at 0.25, 0.5, 1, 2 and 4-h after eggs were submerged. The number of eggs hatched at each time point was determined by counting 1^st^ instars, which were not removed from the watch glass. Eggs in the control R2A medium were monitored for hatching for 24-h and eggs in sterile distilled water were monitored for 5 days. After 5 days, eggs were tested for hatchability by submerging them in a 24-h old bacterial culture. Unless otherwise specified, egg hatch experiments were replicated 5 times for each infusion or cell suspension using different batches of eggs collected on different dates.

### Preparation of white oak leaf infusions

In previous research [16], [17], we established that microorganisms in white oak leaf (WOL) (*Quercus alba*) infusion elicited oviposition responses of *Ae. aegypti*. To determine the functional role of microorganisms in egg hatch, non-sterile WOL infusions were prepared by fermenting senescent leaves (4.2 g) in well water (1 L; 4.2 mg/mL) in sterile glass jars (2 L) fitted with threaded plastic lids [17]. Similarly, sterile WOL infusion was prepared in sterilized glass jars (2 L) by combining sterilized leaves and sterilized well water (autoclaved for 45 min at 120°C). Jars were held at 28°C for one week before infusions were tested in egg hatching experiments. To evaluate effects of water-extractable leaf compounds in stimulating egg hatch, WOL infusion was prepared using senescent leaves that had been ground into fine particles with a commercial blender (Waring Products, Inc., Torrington, CT, USA). WOL powder (2.1 g) was added to a 2 L flask containing 0.5 L sterile water (4.2 mg/mL). The flask was shaken on a rotary shaker at 200 rpm for 20 min at 28°C. After shaking, the WOL infusion was poured through a fine mesh nylon screen to remove leaf particles and the resulting WOL extract was subjected to ultra-filtration (sterile 0.22 µm filter membrane, 47 mm dia., Millipore, Billerica, MA, USA) to remove microbes. The sterile filtrate containing WOL compounds was tested in egg hatching experiments.

To further evaluate the role of microorganisms in stimulating egg hatch, 10 mL of non-sterile WOL infusion was filtered through a sterile 0.22 µm filter membrane (47 mm dia., Millipore, Billerica, MA, USA) to remove microbes. The filtered microbes were then re-suspended in 10 mL sterile WOL infusion. This suspension of microbes was evaluated in egg hatching bioassays. Sterile infusion and sterile water were used as control hatching media for these experiments.

### Bacterial isolates, culture conditions, and preparation of bacterial cell suspensions

To investigate effects of bacteria in stimulating egg hatch, we used a mix of 14 bacterial species, originally isolated from an infusion made from the senescent leaves of canebrake bamboo (*Arundinaria gigantea*); this mix is highly effective at stimulating gravid females to oviposit [16]. A list of the 14 species in the mix of bacteria used in egg hatching experiments is provided in the supporting information (Table S1). Bacterial cultures of each of the 14 species were grown overnight in R2A medium, diluted to 10^4^ cells/mL, and 100 µL of the cell suspension of each species was added to 100 mL of fresh R2A medium. Cultures of this 14-species mix were grown for 24-h in R2A medium (pH 7.2) in Erlenmeyer flasks (250 mL) and aerated on a rotary shaker at 120 rpm at 28°C. Bacterial abundance was estimated by counting colony forming units (CFUs) after spread-plating on R2A agar plates [18] or with a hemocytometer, as described below. To determine if egg hatching cues were metabolic by-products excreted by bacteria into the culture medium, 10 mL of 24-h old cultures were filtered through a sterilized 0.22 µm filter membrane (Millipore) to remove bacteria, and the filtrate was tested in egg hatching bioassays. Effects of bacterial cells were also tested separately. Bacterial cells were harvested from 24-h old cultures by centrifugation at 1,254× g for 10 min and the pellet was suspended in saline solution (0.85% NaCl). The cell suspension was re-centrifuged, the pellet was re-suspended in saline and diluted with saline to produce a dilution series of 10^9^, 10^8^ and 10^7^ cells/mL. These cell densities were tested in egg hatching experiments. A pivotal feature of this dose-response design was that we expected bacteria in sterile saline solution to be metabolically less active, and thus, by maintaining high DO concentration in the presence of bacteria, we uncoupled the naturally tight linkage between high bacterial density and low DO.

### Measuring bacterial abundance and dissolved oxygen

Samples of non-sterile and sterile WOL infusions, bacterial cultures, and filtrate of bacterial cultures were taken for enumeration of CFUs at 0-h (just before eggs were submerged) and 4-h after egg hatching bioassays were initiated. Duplicate subsamples (1 mL each) from each infusion or bacterial culture were mixed separately in 9 mL of 0.1% peptone (in sterile water, wt/vol) and serially diluted (10-fold) in 0.1% peptone. After dilution, 100 µL of each of the 10^−2^ to 10^−5^ infusion dilutions and 10^−5^ to 10^−7^ culture dilutions were spread separately on each of two R2A agar plates, and the plates were incubated at 28°C. To estimate the number of CFUs per mL, colonies were counted on each dilution plate 4–5 d after spread-plating. Total cells in cultures of the mix of 14 bacterial species were counted using a hemocytometer 0, 0.25, 0.5, 1, 2 and 4-h after egg hatching experiments were started. Bacterial cells in control R2A medium were counted at 0, 0.25, 0.5, 1, 2, 4, 8, 16 and 24-h. At the same experimental time points, DO concentrations were measured with an oxygen electrode control unit (CB1-D3, Hansatech Instruments Ltd., Norfolk, England).

### Data analysis

For each replicate, the cumulative number of eggs hatched at each time point was converted to a percentage of the total number of eggs (*n* = 50) at the start of the experiment. The percentage values were subjected to an arcsin (sqrt *x*) transformation to achieve approximate normality. The transformed data for each experiment were separately analyzed as a repeated measures study using a mixed model analysis of variance (PROC MIXED, SAS for Windows, ver. 9.1, SAS Institute, Cary, NC). In these analyses, transformed values for the percentage egg hatch in the test and control media were the dependent variable and, *Treatment* (test or control) and *Time* (0, 0.25, 0.5, 1, 2, and 4-h) were independent categorical variables. Each analysis included a *Treatment* x *Time* interaction with *Replicate* (*Treatment*) included as a random effect and *Treatment* x *Replicate* as the repeated factor. Because test and control eggs were hatched in separate containers, residual variances were separately fit by the model using the GROUP =  *Treatment* option on the REPEATED statement. An LSMEAN statement with a SLICE option was used to construct *t*-tests to test the null hypothesis that the difference in the percentage egg hatch in test and control media at each time point was zero. Analysis of the dilution series of bacterial cell densities was separately carried out for each cell density using the same mixed model.

## Results

### Egg hatch in non-sterile and sterile white oak leaf infusion

**Figure 1:**
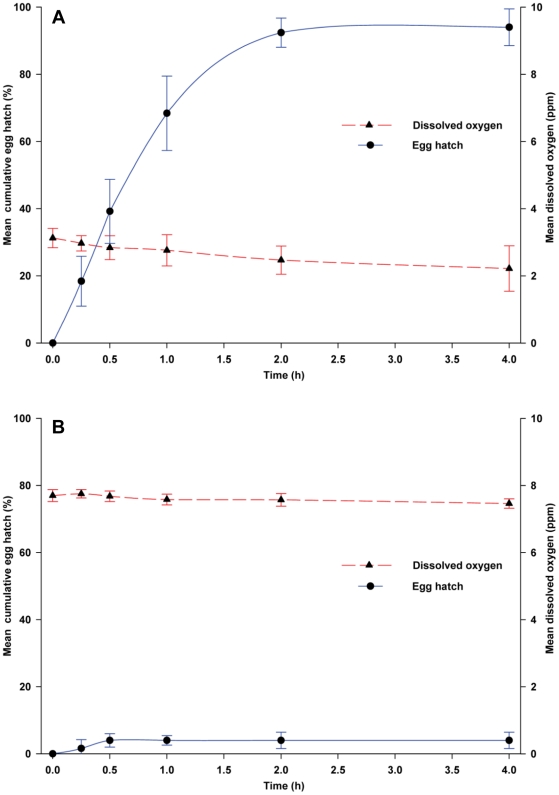
(A) Egg hatching responses of *Ae. aegypti* to non-sterile white oak leaf infusion. (B) Egg hatching responses of *Ae. aegypti* to sterile white oak leaf infusion. Data points are the mean ± SD for 5 replicate experiments, with 50 eggs per replicate.

Sterile distilled water failed to stimulate *Ae. aegypti* eggs to hatch, with only 2.0% hatching after 4-h, and only 4.0% of the eggs hatching after 5 days. In contrast, non-sterile WOL infusion stimulated a rapid and monotonic increase in *Ae. aegypti* egg hatch within 2 h (Fig. 1A). An average of 92.4% of the eggs hatched within 2-h and 94.0% hatched within 4-h in non-sterile WOL infusion. During the 4-h experimental period, DO levels declined from 3 to 2 ppm (Fig. 1A). Colony forming units, measured to characterize the abundance of heterotrophic bacteria in the leaf infusion, were 8.94×10^7^ CFU/mL at 0-h and 5.66×10^7^ CFU/mL at the 4-h time point.

Sterile WOL infusion, produced by combining sterilized oak leaves and sterilized well water, elicited a markedly different response compared to non-sterile WOL infusion. Only 4.0% of the eggs hatched within 0.5-h and no more eggs hatched after 4-h in the sterile infusion (Fig. 1B). Differences in the percentage of eggs hatching over time for the non-sterile and sterile WOL infusions were highly significant (*F* = 105.27; df = 5, 40; *P*<0.0001). Percentage egg hatch in non-sterile WOL infusion for all time points beyond 0-h was significantly greater (*P*<0.0001) than in sterile infusion. Likewise, a microbe-free leaf extract, obtained by ultra-filtration of an infusion of ground WOL steeped in sterile water for 20 min, was minimally bioactive – only 14.8% of eggs hatched after 4-h. No CFUs were detected in the sterile infusion and leaf powder extract that were sampled at 0-h. However, bacterial densities of 5.66×10^4^ CFU/mL and 1.23×10^4^ CFU/mL were measured at the end of the 4-h period in autoclaved infusion and WOL extract, respectively. Over the 4-h period, DO concentration in the sterile infusion ranged between 7.4 and 7.6 ppm (Fig. 1B) and the DO concentration in the WOL extract ranged between 6.4 and 6.6 ppm.

**Figure 2:**
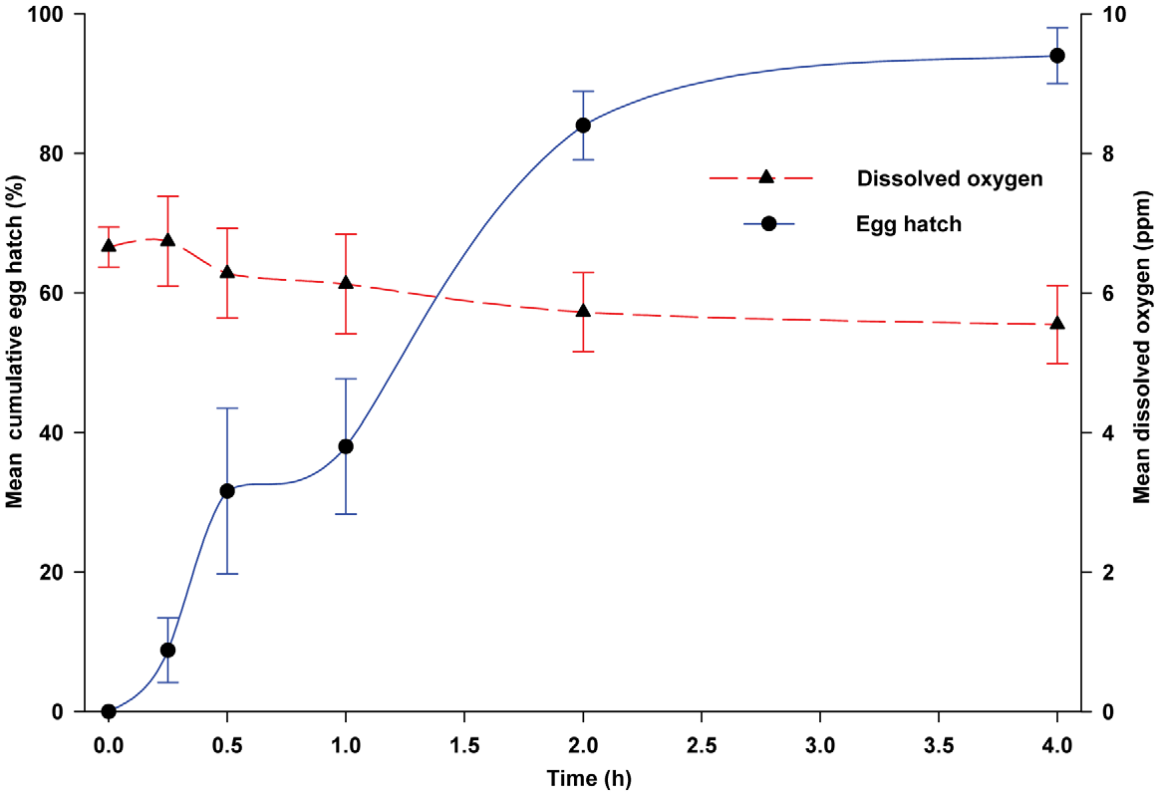
Egg hatching responses of *Ae. aegypti* to microbes resuspended in sterile infusion. Non-sterile WOL infusion was filtered through a sterile 0.22 µm filter membrane and the retained microbes were added to sterile WOL infusion, which by itself did not stimulate eggs to hatch. Data points are the mean ± SD for 5 replicate experiments, with 50 eggs per replicate.

These results suggested to us that either high microbe density (>10^4^ CFU/mL), low DO concentration (<3 ppm), or both, are needed to stimulate egg hatch, and that water-soluble sterile leaf extract fails to stimulate egg hatch. To verify the involvement of microbes in stimulating eggs to hatch, non-sterile WOL infusion was filtered through a sterile 0.22 µm filter membrane and the retained microbes were added to sterile WOL infusion that by itself stimulated only 4.0% of the eggs to hatch. This suspension of microbes stimulated 84.0% of eggs to hatch within 2-h and 94.0% in 4-h (Fig. 2). We found highly significant differences between the percentage of eggs hatching over time in sterile infusion containing the re-suspended microbes and sterile infusion (*F* = 143.97; df = 5, 40; *P*<0.0001). Differences in egg hatch were highly significant (P<0.0001) at each time point. The microbial suspension contained 4.81×10^7^ CFU/mL of culturable bacterial cells at the end of the 4-h period. Notably, over the 4-h exposure period, DO concentration ranged between 6.6 and 5.5 ppm (Fig. 2), indicating that in the presence of microbes, moderately high DO did not inhibit egg hatch.

### Effects of bacterial cultures on hatching of *Aedes aegypti* eggs

**Figure 3:**
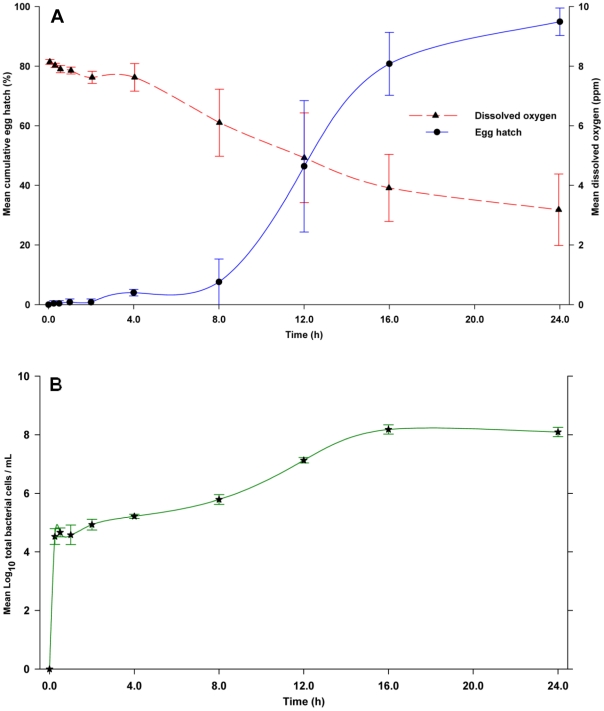
(A) Egg hatching responses of *Ae. aegypti* to a control R2A medium. (B) Variation of mean cell counts at the time of egg hatch. Data points are the mean ± SD for 5 replicate experiments.

Having established that factors stimulating egg hatch are associated with microbes in WOL infusion, we next exposed eggs to either sterile control R2A medium or 24-h old R2A culture of a mix of 14 bacteria species isolated from bamboo leaf infusion. In sterile control R2A medium, the hatching response was low with 4.0% of the eggs hatching after 4-h of incubation (Fig. 3A). However, the percentage of eggs hatching increased markedly to 95.0% after 24-h (Fig. 3A). Bacterial cell density increased to 1.64×10^5^ cells/mL after 4-h, presumably from bacteria associated with the eggs, and further increased over time to 1.24×10^8^ cells/mL at 24-h (Fig. 3B). The DO concentration was 7.62 ppm at 4-h (Fig. 3A), but subsequently declined to 3.18 ppm at the 24-h time point because of the increased microbial activity.

**Figure 4:**
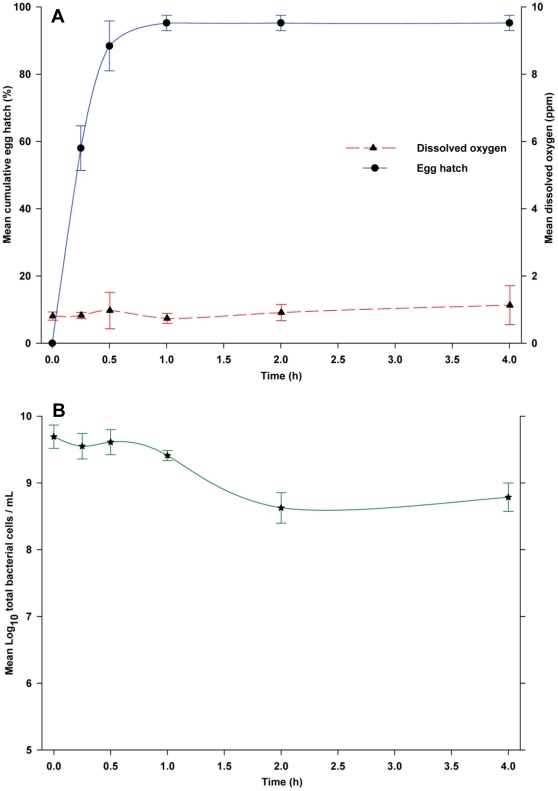
(A) Egg hatching responses of *Ae. aegypti* to a mix of 14 bacterial species isolated from a bamboo leaf infusion and cultured for 24-h in R2A medium. (B) Variation in mean bacterial cell density during the 4-h assay period. Data points are the mean ± SD for 5 replicate experiments.

In contrast, the mix of 14 bacteria stimulated a very rapid response with 88.4% of the eggs hatching within 30 min, and 95.2% within 1-h (Fig. 4A). Densities of total culturable bacteria averaged 3.02×10^9^ CFU/mL and 5.92×10^8^ CFU/mL at 0-h and 4-h, respectively. Differences in the percentages of eggs hatching over time for the mix of 14 bacteria and control medium were highly significant (*F* = 205.86; df = 5, 40; *P*<0.0001). Likewise differences at each time point beyond 0-h were highly significant (*P*<0.0001). Similarly, bacterial densities in direct microscopic counts declined from 4.91×10^9^ cells/mL at 0-h to 6.10×10^8^ cells/mL at 4-h (Fig. 4B), suggesting that the feeding activity of the rapidly hatched larvae was reducing the abundance of bacteria. However, because DO concentration in the culture medium was low, ranging from 0.80 to 1.12 ppm, this experiment could not differentiate effects of microbes and low DO.

### Uncoupling effects of bacteria from DO

**Figure 5:**
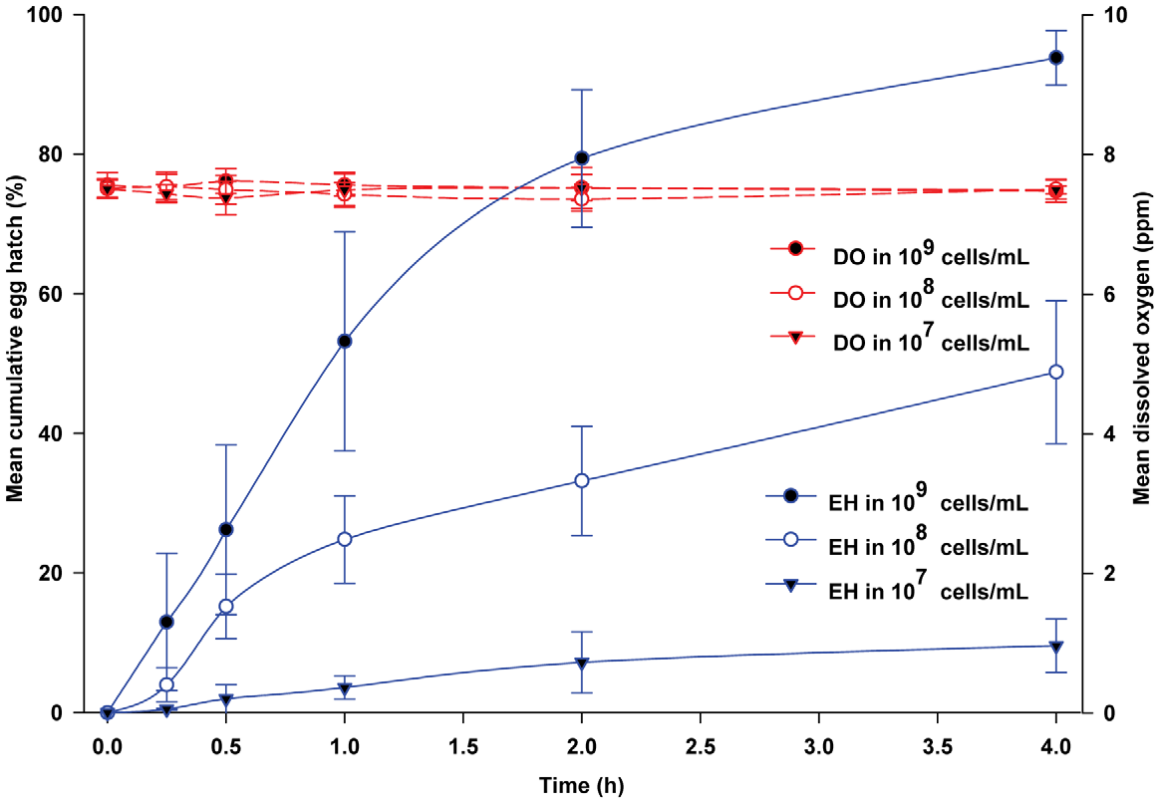
Egg hatching responses of *Ae. aegypti* to a mix of 14 bacterial species harvested from a 24-h old culture by centrifugation and re-suspended in sterile saline. Data points are the mean ± SD for 5 replicate experiments, except 10^9^ cells/mL, which was evaluated with 10 replicate experiments. Abbreviations used in legend as follows: DO, dissolved oxygen; EH, egg hatch.

In the following experiments, our aim was to demonstrate that bacterial cells stimulated eggs to hatch independently of the level of DO. Because eggs hatched in the presence of microbes and moderately high DO (Fig. 2), we used a modification of this design. The mix of bacterial species was recovered by centrifugation from a 24-h culture in R2A, and re-suspended in sterile saline solution to produce a dilution series of 10^9^, 10^8^ and 10^7^ cells/mL. An important feature of this design was that we expected bacteria in sterile saline solution to be much less metabolically active, and therefore DO should be relatively unaffected. *Aedes aegypti* egg hatch exhibited a clear dose-response relationship with greater hatch at higher bacterial density. After 4-h, egg hatch was 93.8% in 10^9^ cells/mL, 48.8% in 10^8^ cells/mL, and only 9.6% in 10^7^ cells/mL (Fig. 5). Differences in the percentage hatch between saline containing bacteria and sterile saline were significant over the 4-h time course at 10^9^ cells/mL (*F* = 78.77; df = 5, 40; *P*<0.0001), 10^8^ cells/mL (*F* = 23.53; df = 5, 40; *P*<0.0001), and 10^7^ cells/mL (*F* = 3.0; df = 5, 40; *P*<0.0217). Only at 2 and 4-h were differences in egg hatching between test and control media statistically significant (*P*<0.001). As expected, DO concentrations were consistently high across the 3 bacterial densities and over the entire 4-h experimental period, ranging from 7.36 to 7.62 ppm (Fig. 5).

**Figure 6:**
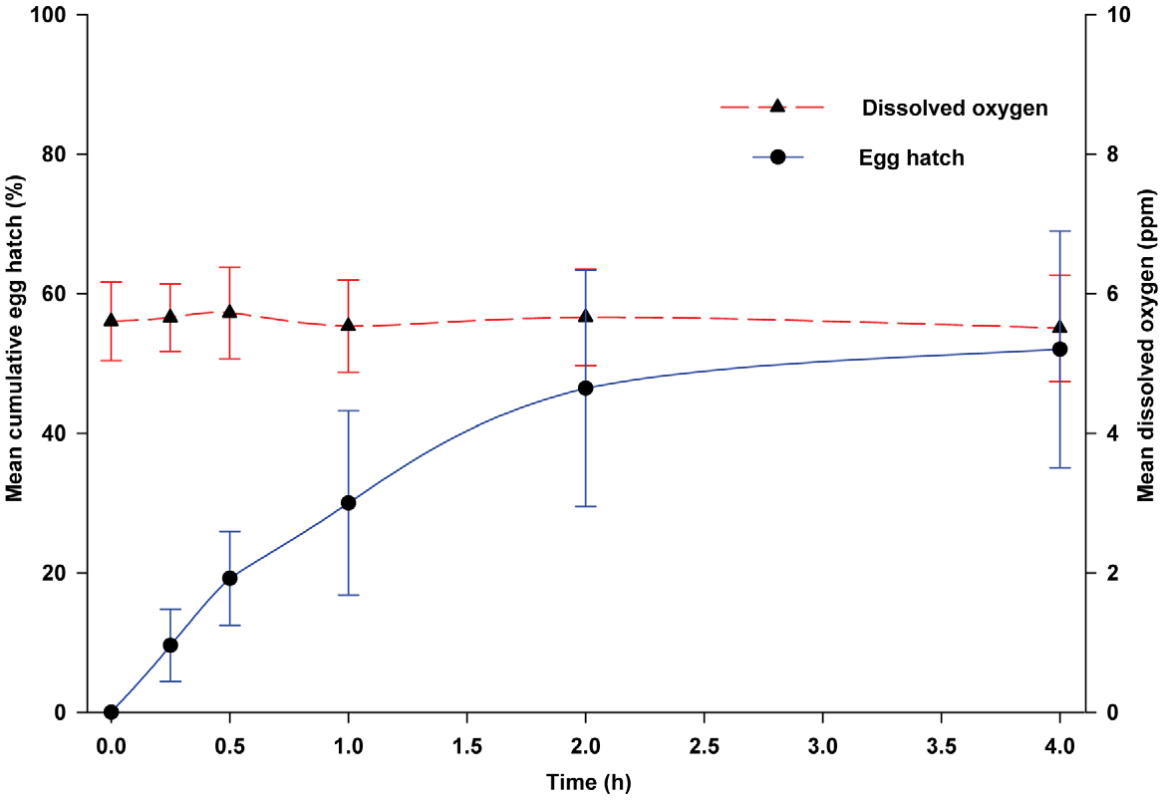
Egg hatching responses of *Ae. aegypti* to bacteria-free culture medium from a 24-h old bacterial culture that was sterilized by filtration through a 0.22 µm filter. Data points are the mean and bars are ± SD for 5 replicate experiments.

Several control treatments were conducted to confirm these results. First, sterile saline solution failed to stimulate any egg hatch, indicating that bacteria on the egg surface did not stimulate hatching under these experimental conditions. In addition, to test the bioactivity of water-soluble bacterial metabolites, we obtained 24-h old bacterial cell-free media by filtering the 24-h old culture through a 0.22 µm filter. This medium stimulated a slow and moderate rate of egg hatch, with only 19.2% hatching within 30-min and 52% after 4-h. Differences for the percentage of eggs hatching over time in water-soluble bacterial metabolites and control R2A medium were highly significant (*F* = 27.16; df = 5, 40; *P*<0.0001). At all time points beyond 0-h, a significantly greater (*P*<0.0001) percentage of eggs hatched when exposed to bacterial metabolites. DO concentration in the culture medium ranged from 5.6 to 5.5 ppm (Fig. 6). Although no bacteria were cultured immediately after filtration, at the 4-h time point the bacterial density was 6.73×10^4^ CFUs/mL. Collectively, these experiments effectively uncoupled effects of microbes and DO, and they conclusively showed that bacteria stimulate egg hatch in *Ae. aegypti* even at high DO concentrations.

## Discussion

Based on the stimulatory effects of reduced DO concentration in the water, Horsfall et al. [19] classified the hatching responses of aedine eggs into three types. Type I eggs hatch after contact with water without any significant decline in DO concentration. Type II eggs are stimulated to hatch when DO levels are reduced, and Type III eggs will not hatch under declining DO conditions until properly conditioned. Results of our experiments provide the basis for a new paradigm of factors that stimulate *Ae*. *aegypti* eggs to hatch. Specifically, biological cues associated with bacteria, independently of low DO, stimulate eggs of *Ae. aegypti* to hatch. To our knowledge, the present study is the first to uncouple effects of DO on egg hatch from effects of factors associated with plant infusions and bacterial cultures. Moreover, we combined these studies with quantitative measurements of bacterial populations, allowing for a direct correlation between bacterial density and egg hatch at high DO concentration.

Several early reports associated plant infusions and bacterial cultures with egg hatch in mosquitoes. However, the causative relationships among bacterial cell density, DO concentration, and water soluble microbial metabolites remained ambiguous. For example, Gjullin et al. [7] showed that 80% of eggs of *Ae. lateralis* (Meigen) (presumed to be *Ae*. *sticticus* (Meigen)) and *Ae. dorsalis* (Meigen) hatched in 1 to 2-h in infusions made from willow leaves in tap water. Although microbes were clearly implicated, bacteria were not cultured, DO was not measured, and this work concentrated more on candidate organic compounds that could stimulate hatching. Use of bacterial cultures was first reported by Atkin and Bacot [5], and although subsequent papers noted that egg hatching occurred concurrently with microbial growth [12], [13], bacterial cell densities were not measured. In our experiments, submersion of *Ae. aegypti* eggs in non-sterile WOL infusion for 4-h resulted in high hatch rates (92.4%) at relatively low DO levels (2–3 ppm), and a bacterial cell density of 5.6×10^7^ CFU/mL was cultured at the end of the exposure period. Likewise, we found that an average of 95.2% of eggs hatched within 1-h of submersion in a culture composed of 14 bacterial species with a DO concentration ranging from 0.73 to 0.84 ppm. Because a bacterial density of 1.64×10^5^ cells/mL failed to stimulate egg hatch, we suspected that higher cell densities were required to elicit this effect.

However, unless experimentally manipulated, an increase in bacterial density is always accompanied by a decline in DO. Several investigations measured this decline in DO concurrently with egg hatch of aedine species [8], [12], [20], and generally concluded that low DO – produced physically, by microbial growth or with reducing agents – constituted an important hatching stimulus. A slow decrease in oxygen concentration was observed to be more effective in stimulating hatching of *Ae. aegypti* eggs than was an initially low and static oxygen concentration [14]. We employed two complementary approaches to maintain relatively high DO levels in the presence of bacteria. In the first approach, we filtered bacterial cells from a WOL infusion and added the filtered microbes to sterile WOL infusion. This assay produced extraordinarily rapid egg hatching (84.0% within 2-h and 94.0% in 4-h), and moderate microbial density (4.81×10^7^ CFU/mL), while maintaining relatively high DO concentrations of 5.5–6.6 ppm. In our second approach, we manipulated the initial bacterial cell density, and by re-suspending bacteria in sterile saline solution we were able to maintain relatively high DO levels throughout the 4-h assay. These dose (cell density)-response (egg hatch) experiments showed that 93.8% of eggs hatched after 4-h exposure to 10^9^ bacterial cells/mL at a high DO concentration of 7.5 ppm. Notably, at lower cell densities of 10^8^ cells/mL and 10^7^ cells/mL, egg hatch declined to 48.8% and 9.8%, respectively. These are the clearest results to date showing that bacteria or bacterial metabolites alone can stimulate rapid egg hatch in *Ae. aegypti*, with no need for a concomitant decline in DO concentration.

Our results also provide evidence for the involvement of water-soluble bacterial metabolites in stimulating eggs to hatch. First, the experiments with bacterial cells suspended in sterile saline solution required higher bacterial cell densities to achieve significant egg hatch than when bacteria were suspended in R2A medium. We suspect that this was because the bacterial cells were not metabolically active in saline solution. Furthermore, bacteria cell-free metabolites obtained through ultra-filtration of bacterial cultures stimulated 52.0% of the eggs to hatch in 4-h, indicating that some water-soluble bacterial metabolites act as hatching stimulants. These results provided the basis for our hypothesis that both bacteria and bacterial metabolites stimulate *Ae*. *aegypti* eggs to hatch. It should be noted that these experiments were conducted with a mix of 14 species. Which of the 14 bacterial species stimulate *Ae*. *aegypti* egg hatching has not been determined.

The bacteria that stimulated egg hatch in our assays originated from leaf infusion or bacterial cultures. However, some bacteria may be introduced with deposited eggs. Gillett el al. [9] and Edgerly and Marvier [10] found that bacteria on the surface of the eggs are involved in egg hatching. In our investigation, few eggs (3.0–4.0%) hatched over a 4-h period in sterile control media (WOL infusion, R2A medium or distilled water). Although these media were sterile at the 0-h time point, 4-h later they contained approximately 10^4^–10^5^ bacterial cells/mL. Bacterial cells from the surface of the eggs thus inoculated the sterile medium, as might be expected when *Ae. aegypti* oviposit in human-made water-filled containers. Nevertheless, this low density of bacteria was insufficient to trigger substantial egg hatch, suggesting that a longer incubation period would be required under these conditions. We are presently determining the threshold density of bacteria needed for stimulating significant hatch of *Ae*. *aegypti* eggs.

Taken together, results of these experiments indicate that cues stimulating *Ae. aegypti* eggs to hatch were associated with microorganisms in plant infusions, and could activate hatching independently of a decline in DO. Because previous investigators successfully stimulated eggs to hatch with physical and chemical agents that produced low DO levels, it is expected that low DO can also activate hatching independently of other egg hatching stimuli. Additionally, plant-derived chemicals could also stimulate eggs to hatch, independently of either low DO or microorganisms, but our results downplay the importance of such plant-derived chemical mediators of egg hatch. In support of a role for plant-derived chemicals in mosquito egg hatching, it was reported that amino acids and proteins commonly present in vegetation stimulated eggs of *Ae*. *vexans* and *Ae*. *aldrichi* ( =  *Ae*. *sticticus*) to hatch [7]. Furthermore, Horsfall [21] showed that glucose and the plant growth hormone indole-3-acetic acid were stimulating factors for hatching *Psorosphora discolor* (Coquillett) eggs. In contrast, however, Abdel-Malek [22] reported that eggs of *Ae. trivittatus* (Coquillett) hatched erratically in dilute solutions of plant growth regulators such as *α*-naphthaleneacetic acid, indole-3-acetic acid, and indole-3-butyric acid. In our experiments, plant infusions were sterilized by autoclaving. Consequently, it is possible that using high temperature to kill microbes degraded biochemicals in WOL infusion. However, the microbe-free leaf extract that we obtained by ultra-filtration of an infusion of ground WOL steeped in sterile water for 20 min caused only 14.8% of eggs to hatch during a 4-h of exposure. Unquestionably, egg hatching stimulated by microbes is of much greater significance than plant chemicals. However, there are clearly bioactive compounds in leaf infusion that stimulate a significant percentage of eggs to hatch, and future studies should identify these compounds and examine whether maximal egg hatch is stimulated through their interactions with microbes and DO level.

In conclusion, results of our experiments form the conceptual basis for a new model for the biological processes involved in stimulating hatching of *Ae*. *aegypti* eggs. The mechanism(s) involves factors that are associated with microbes in the egg-laying habitats of container-inhabiting mosquitoes. A challenge for the future will be to identify microbial factors that underlie the stimulation of egg hatching.

## Supporting Information

Table S1 List of bacterial isolates used in this study.
(DOC)
