# Supplementary material for: Bacteria Stimulate Hatching of Yellow Fever Mosquito Eggs
Source: PLoS One. 2011 Sep 6;6(9):e24409. doi: 10.1371/journal.pone.0024409 (PMC3167859; doi:10.1371/journal.pone.0024409)
Supplement: Table S1 — List of bacterial isolates used in this study. (DOC) [file pone.0024409.s001.doc]

Table S1. List of bacterial isolates^1^ used in this study.

| Isolate | Accession number in GenBank | Related microorganism (% sequence identity) | Phylogenetic affiliation |
| --- | --- | --- | --- |
| B1 | EU341308 | *Bacillus thuringiensis* (99) | *Firmicutes* |
| B2 | EU341309 | *Enterobacter asburiae* (98) | *Gammaproteobacteria* |
| B3 | EU341310 | *Enterobacter cancerogenus* (98) | *Gammaproteobacteria* |
| B4 | EU341311 | *Pseudomonas fulva* (99) | *Gammaproteobacteria* |
| B5 | EU341312 | *Lactococcus lactis* (99) | *Firmicutes* |
| B6 | EU341313 | *Enterobacter gergoviae* (97) | *Gammaproteobacteria* |
| B7 | EU341314 | *Enterobacter ludwigii* (97) | *Gammaproteobacteria* |
| B8 | EU341315 | *Klebsiella oxytoca* (98) | *Gammaproteobacteria* |
| B9 | EU341316 | *Klebsiella granulomatis* (98) | *Gammaproteobacteria* |
| B10 | EU341319 | *Pseudomonas plecoglossicida* (99) | *Gammaproteobacteria* |
| B11 | EU341318 | *Rhizobium huautlense* (97) | *Alphaproteobacteria* |
| B12 | EU341319 | *Shigella dysenteriae* (76) | *Gammaproteobacteria* |
| B13 | EU341320 | *Citrobacter freundii* (97) | *Gammaproteobacteria* |
| B14 | EU341321 | *Brevundimonas vesicularis* (98) | *Alphaproteobacteria* |

^1^Procedures used to isolate, culture and identify bacterial species are described in Ponnusamy et al. [16].
